# Screening, Monitoring, and Treatment of Precancerous Atrophic Gastritis in the Prospective Study for Seven Years

**DOI:** 10.31557/APJCP.2020.21.2.331

**Published:** 2020

**Authors:** Sergey M Kotelevets, Sergey A Chekh

**Affiliations:** 1 *Department of Therapy, Medical Institute, *; 2 *Department of Software Development, Department of Mathematics, Institute of Applied Mathematics and Information Technology, North Caucasus State Academy for Humanities and Technologies, Cherkessk, Stavropolskaya Street 36, Russian Federation. *

**Keywords:** Screening, atrophic gastritis, risk of gastric cancer, gastrin-17, pepsinogen-1

## Abstract

**AIM:**

Develop a program to identify, treat, and prevent severe atrophic gastritis to reduce gastric cancer incidence and mortality.

**MAterials and Methods:**

In total, 2,847 people aged > 40 years old underwent serological noninvasive screening for atrophic gastritis by identifying postprandial gastrin-17 and pepsinogen-1 in the fasting state. Anti-H pylori IgG was found in 2,134 patients. Seven years later, 2,220 patientswho had undergone serological noninvasive screening were asked to fill out a questionnaire survey (were interviewed). We could not find any information on 627 of 2,847 patients. Next, 75 patients with multifocal atrophic gastritis who underwent gastroscopy and biopsies (the Updated Sydney System (USS)) were selected. To study gastrin-17 production, morpho-functional correlation was studies in 75 patients with multifocal atrophic gastritis.

**Results:**

During seven years, no reported case of gastric cancer was done among 2,220 persons who underwent serological screening and treatment. In the same population, 4.3 persons who did not receive screening during the same period, developed gastric cancer and died of it. In this study, we can say that 4.3 lives were saved out of 2,220 tested persons. The cost for screening this number of people amounted to €23,750. A comparison of the prevalence rate of the four stages of multifocal atrophic gastritis based on the data of the histopathology tests and noninvasive serologic screening in accordance with OLGA classification showed a strong correlation (the correlation coefficient is 0.812). This finding suggested that using this classification not only for histopathology tests for atrophic gastritis but also for serologic markers of antral mucosa and corpus ventriculi atrophy: gastrin-17 and pepsinogen-1.

**Conclusion:**

Complex pathogenetic treatment of atrophic gastritis significantly reduced gastric cancer risk and incidence for such patients.

## Introduction

It is possible to reduce stomach cancer mortality only in case of early detection. For the modern diagnostics of early stomach cancer, endoscopic monitoring of the stomach’s mucous membrane is required in patients with severe atrophic gastritis (Sugano et al., 2015; Choi, 2014). Many authors have suggested a method for serologic screening of atrophic gastritis based on the levels of substances produced by the antral mucosa and corpus ventriculi in blood serum (Kikuchi et al., 2011; Lijima et al., 2009; Germaná et al., 2005; Vaananen et al., 2003; Storskrubb et al., 2008). Evaluation of serologic markers for atrophic gastritis, such as gastrin-17, pepsinogen-1, pepsinogen-2, the pepsinogen-1/pepsinogen-2 ratio was developed by Biohit; however, this evaluation did not allow us to determin the degree of stomach mucous membrane atrophy in cases of atrophic gastritis. It is critical to identify severe atrophic gastritis during screening as it is considered a precursor condition for gastric cancer. Many studies have shown poor sensitivity for serological assessment of atrophic gastritis because it does not account for the degree of antral mucosa and corpus ventriculi atrophy. It is incorrect to perform comparative studies on ranked histologic criteria with non-ranked serologic criteria. The use of a specific serologic criterion for the gastrin-17 antral mucosa atrophy marker is important. For instance, the gastrin-17 level of 5 pmol/l does not characterize the antrum ventriculi status. However, this level is used in the GastroSoft diagnostic algorithm (Leja et al., 2011; Pasechnikov et al., 2014). There is a functional relationship between the degree of antral mucosa atrophy and gastrin-17 level and between the degree of corpus ventriculi atrophy and pepsinogen-1 level. The higher the antral mucosa and corpus ventriculi atrophy is, the lower the levels of gastrin-17 and pepsinogen-1 will be (Agréus et al., 2012). Therefore, researchers keep studying the suggested atrophic gastritis serologic markers and searching for more accurate criteria to evaluate serologic screening results (Miki, 2011; Shikata et al., 2012; Huang et al., 2015; Roman et al., 2016). S.M. Kotelevets and S.A. Chekh developed and suggested more accurate, highly sensitive criteria to evaluate atrophic changes in antral mucosa and corpus ventriculi. These criteria of the serologic markers of atrophic gastritis allowed determining the degree of antral mucosa and corpus ventriculi atrophy. These criteria were developed based on a thorough analysis of morpho-functional comparisons with histologic criteria. Serologic and histologic criteria of atrophy markers were ranked by the degree of atrophic changes in the gastric antrum and corpus. This approach allowed for consistent comparisons based on calculating the Spearman rank correlation coefficient (Kotelevets and Chekh, 2015). The authors also noted the good quality of the Biohit method for the gastrin-17 and pepsinogen-1 level identification in blood.

Research purpose and objectives; Perform serological screening for atrophic gastritis using the atrophy marker evaluation criteria suggested by S.M. Kotelevets and S.A. Chekh, followed by treatment and long-term monitoring of patients suffering from this disease, and assess the advantages of the suggested method.

## Materials and Methods

In 2010, 2847 persons in the Karachay-Cherkess Republic underwent serological noninvasive screening for atrophic gastritis. To do so, GastroPanel^®^[Finland Biohit Plc, Helsinki] assay kit was used to identify parameters of postprandial gastrin-17 and pepsinogen-I in the fasting state. Anti-H. pylori IgG was found in 2,134 persons. These individuals underwent serological screening after preliminary clinical examination. Written informed consent was obtained from each of the patient. Determination of mild, moderate, or severe atrophy of the gastric mucosa in the stomach antrum and corpus was determined based on the criteria developed by S.M. Kotelevets and S.A. Chekh. In 2017, seven years later, 2,220 persons, who had undergone serological noninvasive screening for atrophic gastritis in 2010, were asked to fill out a questionnaire (were interviewed). In 2017, the researchers failed to find any information about 627 out of 2,847 persons who had undergone serological screening in 2010. During 7 years, 627 persons were excluded for the following reasons: died of cardio-vascular diseases, moved to another region, refused to participant in the study, or were absent for unknown reasons. Out of 627 patients, 33 ones died of cardio-vascular diseases. No case of gastric cancer was reported.

Finally, 2,220 patients (2,847 – 627 = 2,220) were included in the current study as the experimental group. All 2,200 patients aged > 40 years old.

To form the control group, we randomized the population of the Karachay-Cherkess Republic, aged 40+. In 2010, 215,412 persons aged > 40 years old were living in this region of the Russian Federation. The control group (which had not undergone serological screening) was formed out of those people. In both groups, the same number of persons was included, 2,220 persons in each group. In 2010, a total of 477,403 persons lived in the Karachay-Cherkess Republic according to the 2010 population census (Status of Oncological Aid to the Population in Russia (2010–2017).

The third group included 75 patients with multifocal atrophic gastritis. They underwent gastroscopy and biopsies (the Updated Sydney System (USS)) based on the results of endoscopic screening. To study the gastrin-17 product, morpho-functional correlations were performed for patients with multifocal atrophic gastritis.

*Statistical analysis*

To determine the proportion of patients with intestinal metaplasia and dysplasia, we calculated the percentage in three groups of patients. The threshold p value of ≤0.05 was set as statistical significance. Given these criteria, differences were identified among three groups. To assess the relationship between the stage of multifocal atrophic gastritis and other parameters, we used nonparametric Spearman correlation coefficient. To assess the results of prevention, seven years later, we determined the number of cases of stomach cancer and the number of saved lives in the experimental and control groups. 

## Results

In the third group, patients with multifocal gastritis whose gastrin-17 level was lower than 10 pmol/l and/or pepsinogen-1 level was lower than 25 µg/l, atrophy in the antral mucosa and corpus ventriculi was confirmed by performing histopathology test of biopsy materials. Biopsy materials were obtained from all patients in strict compliance with the Sydney system. In 67 patients with multifocal atrophic gastritis, gastrin-17 was < 10 pmol/l. In other words, gastrin-17 was reduced in 67 patients according to the criteria posed by S.M. Kotelevets and S.A. Chekh. Gastrin-17 was 10.5 pmol/l in two patients, was 11 pmol/l in three of them , and was 11.5 pmol/l in two of the patients with multifocal atrophic gastritis. In other words, the serum level was close to low in these patients. In one patient, gastrin-17 was 19 pmol/l though this serum level of gastrin-17 is not considered high. For gastrin-17, the sensitivity in this case was 89 %. The results of our study completely refuted the possibility of increased gastrin-17 production in response to stomach corpus mucosa atrophy in cases of concurrent antrum atrophy.

Depending on the degree of atrophy in the antrum and stomach corpus, these 75 patients were classified according to the OLGA system. Distribution under this classification was performed based on histologic study of biopsy materials by USS visual analogue scale. In addition, these patients were classified under OLGA system (Rugge et al., 2007; Rugge et al., 2011; Rugge et al., 2014) based on serologic criteria of atrophy in the stomach antrum and corpus developed by S.M. Kotelevets and S.A. Chekh.

We identified a strong correlation as a result of a nonparametric correlation analysis of the dependency between the diagnostics of a stage of multifocal atrophic gastritis in each of the 75 patients based on histopathology tests and serologic markers pursuant to OLGA classification. The Spearman rank correlation coefficient was 0.725 (n=75). The dependency of histologic and serologic methods of atrophic gastritis stage diagnostics is statistically significant (p<0.001). This finding suggested that using this classification not only based on histologic study of biopsy materials at various stages of atrophic gastritis but also on serologic markers of antral mucosa and corpus ventriculi atrophy, i.e., gastrin-17 and pepsinogen-1 (Table 1).

As a result of a histopathology test of biopsy materials, we detected intestinal metaplasia in 47 out of 75 patients with multifocal atrophic gastritis (63 %). Among these 47 patients, 22 ones had mild intestinal metaplasia (29 %), 15 had moderate intestinal metaplasia (20 %), and 10 (14 %) had severe intestinal metaplasia.

Fewer cases of dysplasia were found in this group. According to histopathology tests, 39 (52 %) patients had dysplasia with various degrees. Out of these patients, 24 patients (32 %) had mild dysplasia, 13 patients (17%) had moderate dysplasia, and 2 patients (3 %) had severe dysplasia.

To identify the correlation between the degree of atrophy and the severity of mucosa dysplasia, the Spearman’s rank-order correlation coefficient was determined. In terms of correlation between degree of atrophy in the antrum and severity of mucosa dysplasia, the coefficient was 0.320 (n = 75, p = 0.005). In terms of correlation between degree of atrophy in the stomach corpus and severity of mucosa dysplasia, the correlation coefficient was 0.235 (n = 75, p = 0.042). To assess total atrophic gastritis severity pursuant to OLGA classification, we determined multifocal atrophic gastritis stages for each patient. As a result, , a stronger relation was found between the multifocal atrophic gastritis stage and severity of stomach dysplasia. The correlation coefficient in this case was 0.41 (n = 75, p < 0.001). It is noteworthy to mention that the lack of strong correlation does not contradict increased risk for gastric cancer following the increase of atrophy degree and dysplasia severity. Atrophy always precedes dysplasia, and these two processes develop at different times; whereas, correlation better describes concurrent processes. Nevertheless, we believe that the performed analysis related to the connection of these two processes may be deemed sufficiently correct .

The results of serologic screening of 2847 persons for atrophic gastritis in 2010 and the results of interviewing the same patients seven years later, pursuant to OLGA classification, are presented in Tables 2 and 3. We identified 504 patients (23 %) with severe antral mucosa atrophy and 10 ones (0.5 %) with severe atrophy of the corpus ventriculi mucosae. Twelve patients were diagnosed with fourth stage atrophic gastritis. Detailed information about all 12 patients diagnosed with fourth stage atrophic gastritis was obtained seven years later, and no case of gastric cancer was reported.

Among patients who withdrew from the study seven years later, no one was diagnosed with fourth stage atrophic gastritis; therefore, the risk of developing gastric cancer among those who withdrew was minimal.

Out of 2,134 patients who were tested for *Anti-H pylori* IgG, 1600 patients had a positive response to *H. pylori *(75 %).

One-week *anti-H. pylori* therapy was assigned to all *H. pylori*-positive patients. Per day, they took 40 mg omeprazole, 1,000 mg clarithromycin, and 2,000 mg amoxicillin. The patients, whose serologic screening revealed severe antral mucosa ventriculi atrophy, were assigned prolonged courses of atrophic gastritis replacement therapy with abomin (1 tablet contained 50,000 units of rennet received from baby calves and lambs, produced by Semashko Moskhimpharmpreparaty, Russia). In case of severe mucosae corpus ventriculi atrophy, patients were given acidin–pepsin. The period of treatment was at least six months within one year. Such replacement therapy courses are recommended to be repeated annually. During the 2017 interview, we found that the patients repeated the replacement therapy courses only in case of dyspepsia symptom aggravation. Patients with severe antral mucosa and corpus ventriculi atrophy (746 persons) were recommended to undergo gastroscopy every year. In fact, 923 patients underwent gastroscopy. In total, 219 patients decided to undergo gastroscopy on their own initiative. Out of 746 patients who were recommended to undergo gastroscopy, 42 patients did not do it for seven years.

Among 2,220 patients who aged > 40 years old and underwent serological screening in 7 years, no case of gastric cancer development was found. It is noteworthy to mention that among the patients who withdrew from the observation (627 patients), there was no patient with moderate or severe atrophic gastritis. In other words, they had a moderate or high risk of developing gastric cancer. Therefore, the risk of developing malignant neoplasm among them during seven years was minimal.

The population of the Karachay-Cherkess Republic was 477,403 in 2010, including 215,412 persons aged > 40 years old. In the second control group for comparison 410 out of 212,565 persons aged > 40 years old were diagnosed with gastric cancer during 7 years (2010–2016), which is equivalent to 4.3 out of 2,220 persons who did not undergo serological screening for atrophic gastritis. During the same seven years, in the first group, no case of gastric cancer was reported among 2,220 persons who had undergone serological screening in 2010. Therefore, we can say that 4.3 lives were saved out of 2,220 screened people. All 12 persons with fourth stage atrophic gastritis under OLGA classification that were found during screening were thoroughly analyzed. Endoscopy findings confirmed that they all had atrophic gastritis. The clinical case of patient P. born in 1952 is of especially great interest. Since 2010, after severe atrophic gastritis (the fourth stage) was found during serologic screening, she underwent systemic treatment and endoscopy for her disease. In 2016, at age 64, she was diagnosed with uterine cancer, from which she died soon after. In six years, during endoscopic monitoring of atrophic gastritis, no progression of disease was found. We may assume that the consistent treatment prevented gastric cancer from developing, regardless of the general reduction of anticancer immunity.

To evaluate financial costs, we calculated the amount of money spent on prevention of one person’s death from gastric cancer. To examine 2,220 persons, 25 sets of Gastropanel kits (gastrin-17, pepsinogen-1 and *anti-H pylori *IgG) produced by the Finnish company Biohit were used. Each one was purchased for RUB 38,000 (in 2010, €1 equaled Ք40). Therefore, Ք38,000 ˟ 25 Gastropanel sets / 40 = €23,750 were spent to test 2220 persons. This amount saved 4.3 lives for 7 years as they avoided gastric cancer development. Hence, to reduce gastric cancer incidence and its mortality within seven years, €23,750 ꞉ 4.3 = €5,523 was spent (labor costs not included).

**Figure 1:**
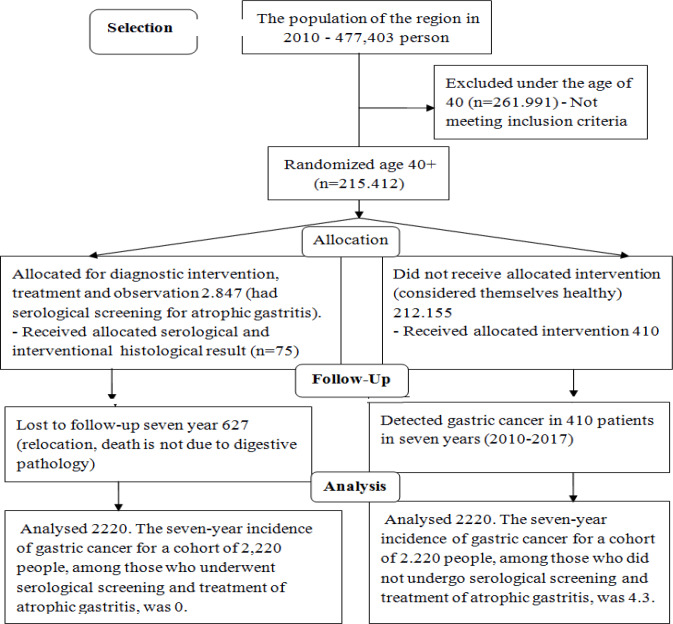
Long-Term Research Results

**Table 1:** Distribution According to OLGA Classification based on Serologic-Histologic Criteria

Serologic criteria	No stomach corpus atrophy Serol-histol	Mild corpus atrophy Serol-histol	Moderate corpus atrophy Serol-histol	Severe corpus atrophy Serol-histol
No antrum atrophy	0 – 0	2 – 0	0 – 0	5 – 0
Mild antrum atrophy	1 – 0	3 – 7	10 – 2	10 – 11
Moderate antrum atrophy	1 – 0	9 – 7	15 – 27	11 – 10
Severe antrum atrophy	0 – 0	2 – 1	3 – 6	3 – 4

**Table 2:** Distribution of OLGA Based on Serology in 2010 and were Interviewed in 2017

Total of 2847 persons-serogy in 2010, total of 2220	No stomach corpus atrophy	Mild corpus atrophy	Moderate corpus atrophy	Severe corpus atrophy
persons-interrogation in 2017 (serogy in 2010)	2010 – 2017	2010 – 2017	2010 – 2017	2010 – 2017
No antrum atrophy	1256 – 992	60 – 43	33 – 28	81 – 60
Mild antrum atrophy	351 – 287	3 – 3	2 – 2	2 – 2
Moderate antrum atrophy	388 – 291	8 – 5	0 – 0	3 – 3
Severe antrum atrophy	641 – 487	10 – 8	4 – 4	5 – 5

**Table 3:** Structure Patients Lost before 2017 (OLGA Classification OLGA based on Serological Criteria)

Total of 627 persons-examined in 2010	No stomach corpus atrophy	Mild corpus atrophy	Moderate corpus atrophy	Severe corpus atrophy
No antrum atrophy	264	17	5	21
Mild antrum atrophy	64	0	0	0
Moderate antrum atrophy	97	3	0	0
Severe antrum atrophy	154	2	0	0

## Discussion

Toyoshima et al., (2017) analyzed the esophagogastroduodenoscopy (EGD) results of 1,232 persons at Toyoshima Endoscopy Clinic in Tokyo, Japan, between April 2002 and June 2014. These patients were diagnosed with atrophic gastritis associated with *H. pylori* infection, and the *H. pylori* infection was successfully eradicated after performing *anti-H. therapy*. During the follow-up period, gastric cancer developed in 15 of 1,232 patients after eradication of *H. pylori* infection (the mean follow-up duration was 2.46 years). The authors believed that gastric atrophy was a major risk factor for gastric cancer development after *H. pylori* eradication. Patients with advanced atrophy should be carefully monitored for at least 10 years (Toyoshima et al., 2017). Wong et al., (2004) found that the incidence of gastric cancer development at the population level was similar between participants receiving *H. pylori *eradication treatment and those receiving placebo during a period of 7.5 years in a high-risk region of China. In the subgroup of *H. pylori *carriers without precancerous lesions, eradication of *H. pylori* significantly decreased the development of gastric cancer. These conclusions are consistent with findings of Kotelevets et al., (2016) who believed that atrophic gastritis and gastric cancer were most likely caused by single nucleotide polymorphisms. *H. pylori* infection is not the only factor contributing to the development of gastric cancer. Atrophic gastritis not associated with *H. pylori* also contributes to the development of gastric cancer. Unfortunately, etiotropic gene therapy of precancerous atrophic gastritis is currently impossible because this treatment method has not yet been developed. We believe that it is possible to develop a pathogenetic treatment. In our research, *anti-H. pylori* and replacement pathogenetic therapies had good effects and resulted in the mitigation of developing gastric cancer risk and its incidence in patients with atrophic gastritis. We speculate that the therapeutic impact could apply to well-known mechanisms of gastric cancer. In addition to *anti- H. pylori* and replacement enzymatic methods of treatment, the methods of anti-reflux, anti-carcinogenic (Acetium), gastrosparing, and antioxidant therapy may be used in this regard. Comprehensive pathogenetic treatment may result in a significant reduction of gastric cancer risk and incidence in patients with atrophic gastritis.

When mucosae antrum and corpus ventriculi atrophy responds to therapy and transformes from severe to mild as a result of treatment, gastric cancer risk will decline from high to low in cases of atrophic gastritis. Therefore, to monitor antral mucosa and corpus ventriculi conditions, a noninvasive method of serologic diagnostics, instead of an annual esophagogastroduodenoscopy, using gastrin-17 and pepsinogen-1 Biohit markers may be applied based on serologic criteria suggested by Kotelevets et al., (2016).

The use of serological screening requires a preliminary analysis of the characteristics of the population and region for the study. Difficult accessibility regions require lightweight screening methods (Curado et al., 2019). You can use computer intelligent systems based on questioning (Mortezagholi et al., 2019). Screening is required in regions with a high risk of gastric cancer (Chen et al., 2019; Zarean et al., 2019). Individual ethnic populations may have a high risk of gastric cancer (Lim et al., 2019). It is necessary to take into account the ethnic factor and the influence of occupational insalubrity when conducting serological screening for atrophic gastritis (Kotelevets et al., 2019). 

In conclusion, it was found that omplex pathogenetic treatment of atrophic gastritis significantly reduced gastric cancer risk and incidence in such patients. Accordingly, it is necessary to perform a comparative study on medications stimulating stomach mucous membrane atrophy regression. Being aware of the curative potential of the medications leading to atrophic gastritis regression will allow for the efficient mitigation of gastric cancer risk for such patients.
